# New Concepts in Brain Networks

**DOI:** 10.3389/fnsys.2012.00056

**Published:** 2012-08-10

**Authors:** Robert Turner, Gabriele Lohmann

**Affiliations:** ^1^Department of Neurophysics, Max-Planck-Institute for Human Cognitive and Brain SciencesLeipzig, Germany

This special issue on “New Concepts in Brain Networks” contains articles that review or propose new approaches for investigating brain connectivity. This topic is highly relevant and has been at the forefront of neuroscientific research in recent years. While univariate techniques dominated fMRI data analysis at the onset of fMRI brain mapping, it is now generally accepted that the human brain is a highly multivariate, and dynamic complex system, and more powerful techniques are needed to describe its function adequately. Clearly, such techniques should reflect the complexity and dynamics of brain function.

This special topic contains seven articles ranging from reviews of the state of the art to novel clinical applications, and discussions of theoretical background.

The paper by Vértes et al. (2011) belongs to this latter category. Vertes et al. find surprising isomorphisms between the human brain and financial markets. The human brain as well as financial markets share network topologies that are non-random, small-world, modular, and hierarchical with fat-tailed degree distributions. However, the authors find that financial markets appear to be more efficient and also more modular than the human brain. One of the merits of this paper is that it opens a path for translating methodologies developed in one domain to applications in other domains.

Hagmann et al. (2012) provide an excellent review of the state-of-the-art in connectomics as a framework for studying the developing brain. Impressive progress has already been made in recent years in the study of structural and functional brain connectivity during maturation. However, the authors also point to several technical challenges that need to be addressed in the coming years. For instance, tractography is still an open issue since it rests on assumptions which are not always met such as local smoothness and coherence of fiber bundles. This work may have distinct clinical relevance in the future if abnormal development of network properties become identifiable by diagnostic tools.

Knösche and Tittgemeyer (2011) review the role of long-range connections for functional segregation as identified by diffusion weighted magnetic resonance (dMRI). In particular, they focus on cortical parcellations based on connectivity profiles that are often characteristic of brain regions. They note that unique parcellations of the entire cortex may not exist, because such parcellations depend on a number of factors such as the required level of detail and perhaps most importantly, the structural criteria underlying such parcellations. Their word of caution is important to keep in mind when dealing with parcellations.

The two following papers combine novel algorithmic approaches with clinical applications. Allen et al. (2011) examine cross-frequency modulation (cfM) in schizophrenia using multi-channel EEG analyzed with a new data analysis approach. Modulation of high-frequency amplitude by low-frequency phase may play an important role in network coordination and functional integration. It is hypothesized that this mechanism may be impaired in schizophrenia and the findings described in this paper suggest that this may indeed be the case. Indeed, the authors found that global cfM was significantly greater in healthy controls than in patients. Furthermore, they report associations of alterations in cfM with genetic polymorphisms. Their findings are based on a novel algorithmic approach in which they decompose cfM results into independent components via ICA. This reduces a very high-dimensional space to a much more manageable space of a few components that are much easier to interpret.

A new algorithm for “resting state” fMRI (R-fMRI) is proposed in the paper by Lohmann et al. (2012). The key idea is to use reproducibility of correlational patterns as an indicator for clinical relevance. Here, fMRI data of a stroke patient monitored over a period of several months is used as an example for a potential domain of application of this method. Brain regions whose correlation patterns change markedly over time may be the ones that are most affected by post-stroke recovery.

A highly sophisticated algorithmic approach is presented by Smith et al. (2012). While the focus of the previous paper was on bi-directional correlations, the focus is now shifted toward directed “causal” connections measured in fMRI. Inference about causality using fMRI data is known to be a challenging problem. Here the authors present a list of six major issues to be addressed when tackling this problem. They propose a new method called “linear dynamic systems for fMRI” (lDSf) and thoroughly discuss these six issues within the framework of their method. The authors remark that considerable future research is needed before effective connectivity can be reliably assessed from fMRI data.

Finally, Kannurpatti et al. (2012) present an interesting finding that in future may help to assess neural activity using fMRI in task non-compliant clinical populations. Specifically, they show that there exists a linear relationship between the amplitudes in R-fMRI and task-based fMRI so that the amplitude of task-based fMRI in one group of subjects may be predicted from another group of subjects who did not even perform the task. This remarkable finding must of course be further validated in future work, but if it is further substantiated by more data it may be of considerable practical relevance.

Overall, this special issue provides an overview of current research on brain networks. This topic is still in its infancy and many new ideas and novel methodologies will be needed in the future.
